# Effect of a Gluten-Free Diet on Cortical Excitability in Adults with Celiac Disease

**DOI:** 10.1371/journal.pone.0129218

**Published:** 2015-06-08

**Authors:** Rita Bella, Giuseppe Lanza, Mariagiovanna Cantone, Salvatore Giuffrida, Valentina Puglisi, Luisa Vinciguerra, Manuela Pennisi, Riccardo Ricceri, Carmela Cinzia D’Agate, Giulia Malaguarnera, Raffaele Ferri, Giovanni Pennisi

**Affiliations:** 1 Department ‘‘G.F. Ingrassia”, Section of Neurosciences, University of Catania, Catania, Italy; 2 Department of Neurology I.C., “Oasi” Institute for Research on Mental Retardation and Brain Aging (I.R.C.C.S.), Troina (EN), Italy; 3 Spinal Unit, Emergency Hospital “Cannizzaro”, Catania, Italy; 4 Gastroenterology and Endoscopy Unit, University of Catania, Catania, Italy; 5 Department of Biomedical Sciences, University of Catania, Catania, Italy; 6 Department“Specialità Medico-Chirurgiche”, University of Catania, Catania, Italy; University of Ottawa, CANADA

## Abstract

**Introduction:**

An imbalance between excitatory and inhibitory synaptic excitability was observed in de novo patients with celiac disease (CD) in a previous study with Transcranial Magnetic Stimulation (TMS), suggesting a subclinical involvement of GABAergic and glutamatergic neurotransmission in asymptomatic patients. The aim of this investigation was to monitor the eventual changes in the same cohort of patients, evaluated after a period of gluten-free diet.

**Methods:**

Patients were re-evaluated after a median period of 16 months during which an adequate gluten-free diet was maintained. Clinical, cognitive and neuropsychiatric assessment was repeated, as well as cortical excitability by means of single- and paired-pulse TMS from the first dorsal interosseous muscle of the dominant hand.

**Results:**

Compared to baseline, patients showed a significant decrease of the median resting motor threshold (from 35% to 33%, *p*<0.01). The other single-pulse (cortical silent period, motor evoked potentials latency and amplitude, central motor conduction time) and paired-pulse TMS measures (intracortical inhibition and intracortical facilitation) did not change significantly after the follow-up period. Antibodies were still present in 7 subjects.

**Discussion:**

In patients under a gluten-free diet, a global increase of cortical excitability was observed, suggesting a glutamate-mediated functional reorganization compensating for disease progression. We hypothesize that glutamate receptor activation, probably triggered by CD-related immune system dysregulation, might result in a long-lasting motor cortex hyperexcitability with increased excitatory post-synaptic potentials, probably related to phenomena of long-term plasticity. The impact of the gluten-free diet on subclinical neurological abnormalities needs to be further explored.

## Introduction

In recent years, a wide spectrum of neurological and psychiatric complications of Celiac Disease (CD) has been described, although most of the studies focused on overt clinical manifestations such as epilepsy, ataxia, peripheral neuropathy, headache, cognitive impairment, depression, anxiety, and schizophrenia [1–3]. However, subtle neurological changes, that do not reach the clinical threshold, have also been reported [4] and might play a role in the pathophysiological mechanisms underlying neurological involvement in CD.

In a previous study aiming to evaluate the effects of the gluten-mediated immune disorder on intracortical circuits, we investigated the profile of cortical excitability of 20 neurologically asymptomatic de novo CD patients by means of Transcranial Magnetic Stimulation (TMS). Compared to healthy controls, a pattern toward a “disinhibition” and “hyperfacilitation” of the motor cortex was observed, suggesting a subclinical involvement of GABAergic and glutamatergic neurotransmission in asymptomatic patients. In particular, it was suggested that the cross-reaction between anti-gliadin antibodies and neuronal antigens, as well as the altered ion levels related to transglutaminase 6-immunoglobulin deposition, might affect the normal balance between excitatory and inhibitory synaptic excitability. Similarly, anti-glutamic acid decarboxylase (GAD) antibodies synthesized within the nervous system might interfere with GABAergic interneurons activity [5].

In this context, most of the studies, although not all [6,7], highlight the potential therapeutic role of the gluten-free diet not only for the typical intestinal manifestations of CD but also for some of the psychiatric and neurological complications, such as depression, anxiety, behavioural symptoms, peripheral neuropathy and cerebellar ataxia [8–12].

The aim of this new study was to assess the impact of the gluten-free diet at the level of TMS, monitoring the neurophysiological changes observed in the same cohort from baseline. We hypothesized that the dietary regimen may modulate the balance between intracortical excitatory/inhibitory circuits.

## Materials and Methods

### Ethics Statement

The study was approved by the ethics committee of the Azienda Ospedaliero-Universitaria “Policlinico-Vittorio Emanuele”, Catania (Italy). Written informed consent was obtained from all participants prior to the participation, in accordance with the Declaration of Helsinki. All assessments were performed in a controlled laboratory environment.

### Subjects and assessment

Of the original cohort of 20 de novo CD patients [5], three of them were no longer eligible (due to lymphoma of the small intestine in one and lack of gluten-free diet adherence in two) and four were lost (three of them refused the TMS re-testing, one was not contactable). Therefore, 13 patients (3 males and 10 females; median age 39 years, interquartile range 24–46) were finally re-evaluated after a median lag of 16 months of gluten-free diet (interquartile range 14–29). At the study entry, all patients, recruited from the Regional Center for Celiac Disease of the University of Catania (Italy), fulfilled the criteria for CD according to the European Society for Paediatric Gastroenterology Hepatology and Nutrition (ESPGHAN) guidelines [13]. According to the international “ACG Clinical Guidelines for the diagnosis and management of Celiac Disease” [14] patients enrolled in the present study underwent periodic visits with both CD specialist and skilled dietician as well as structured survey to evaluate adherence to the gluten-free diet [15]. Diet compliance was adequate in all patients. Table 1 summarizes the main clinical data and the serological findings of CD patients at follow-up. Major neurological disorders, acute medical illness or organ failure, head trauma, epilepsy, Mini-Mental State Examination (MMSE) score <24 [16], alcohol or drug abuse, use of drugs affecting cortical excitability (i.e. benzodiazepines, mood stabilizers, antipsychotics) and any condition precluding TMS execution were exclusion criteria.

**Table 1:** Clinical-serological features of CD-patients before and after the gluten-free diet.

	Baseline		Follow-up		Weightchange (kg)
	*Ab*	*Comorbidities*	*Ab*	*Comorbidities*	
1	EMA, tTG	iron-deficiency anemia	-	none	0
2	EMA, tTG	iron-deficiency anemia	-	none	+7
3	EMA, tTG	thyroiditis	-	thyroiditis	+4
4	EMA, tTG	asthma	-	none	-3
5	EMA, tTG	thyroiditis	-	thyroiditis	0
6	EMA, tTG	thyroiditis, iron-deficiency anemia	EMA, tTG	thyroiditis, iron-deficiency anemia	+1
7	EMA, tTG	vitiligo	EMA, tTG	vitiligo	+4
8	EMA, tTG	iron-deficiency anemia, thyroiditis, infertility	EMA, tTG	iron-deficiency anemia, thyroiditis, osteopenia	0
9	-	osteopenia	-	iron-deficiency anemia, osteopenia	0
10	EMA, tTG	none	EMA, tTG	none	+8
11	EMA, tTG	none	EMA, tTG	none	+3
12	EMA, tTG	none	EMA, tTG	none	+1
13	EMA, tTG	thyroiditis, iron-deficiency anemia	EMA, tTG	thyroiditis, iron-deficiency anemia	0

Ab: antibodies; CD: celiac disease; tTG: tissue transglutaminase antibodies; EMA: endomysial antibodies;—: negative.

The patients underwent the same assessment performed at the entry of the previous study [5], including clinical-demographic evaluation (age, gender, education, handedness, social and living conditions, general and neurological examinations, co-morbidities), and neuropsychological tests: Mini-mental state examination (MMSE), Structured Clinical Interview for DSM-IV Axis I Disorders (SCID-I) and 17-items Hamilton Depression Rating Scale (HDRS) [17]. Electroencephalogram (EEG) and brain computed tomography scan were not repeated given that the main objective was to screen CD patients for the presence of cerebral calcifications and to rule out evident neuroradiological lesions; at baseline, all the patients had normal scans. Moreover, none of the participants complained of neurological symptoms and general and neurological exams did not show other findings with respect to the first examination.

### Transcranial magnetic stimulation

TMS was performed using a High-power Magstim 200 magnetic stimulator (Magstim Co., Whitland, Dyfed, UK). A 70 mm figure-of-eight coil was held over the motor cortex at the optimum scalp position to elicit Motor Evoked Potentials (MEPs) in the contralateral First Dorsal Interosseous (FDI) muscle of the dominant hand, according to the Edinburgh Handedness Inventory [18]. Electromyographic (EMG) activity was amplified using a Medelec Synergy system (Oxford Instruments) and recorded from silver/silver-chloride surface active electrode, placed over the muscular belly of the target muscle (FDI), with the reference electrode placed distally at the metacarpo-phalangeal joint of the index finger and the ground on the dorsal face of the wrist. Motor responses were amplified and filtered (bandwidth 3–3000 Hz) with gains of 100 μV and 5 mV/div. For the motor nerve conduction study (M and F waves from the FDI muscle), a bipolar nerve stimulation electrode (Medelec Synergy system, Oxford Instruments) with 6-mm diameter felt pads and an inter-electrode separation of 25 mm was used and applied to the ulnar nerve at wrist. Resting motor threshold (rMT) was defined as the lowest stimulus intensity able to elicit MEPs at rest of an amplitude 50 μV in at least 5 of 10 trials, according to the IFCN recommendation [19]. When determining the rMT, TMS started with a subthreshold intensity of stimulation, with the coil placed over the optimal site of stimulation; then, stimulus intensity was gradually increased in steps of 5% of the maximal stimulator output (MSO) until TMS consistently evoked MEPs with peak-to-peak amplitude of more than 50 μV in each trial. Thereafter, stimulus intensity was gradually lowered in steps of 1% MSO until 5 positive responses out of 10 trials were recorded [20]. According to the IFCN practical guidelines, an intertrial interval over 3 seconds (4–6 sec) was used in MEPs measurements [21]. Central motor conduction time (CMCT) was calculated by subtracting the conduction time in peripheral nerves, estimated by F wave techniques, from MEP latency obtained during moderate active muscle contraction, with a stimulus intensity set at 130% of the rMT. The clear negative waveform from the baseline was used to determine the MEPs onset latency. M and F waves are elicited by giving supramaximal electrical stimulation to the ulnar nerve at wrist. The size of the MEPs was expressed as a percentage of supramaximal M wave amplitude (A ratio). The cortical silent period (CSP) was determined with an approximately 50% of maximum tonic voluntary contraction of the FDI muscles, induced by single TMS pulses delivered at 130% of rMT. The mean CSP duration of 10 rectified trials was calculated.

Curves of intracortical excitability were obtained using the paired-pulse TMS protocol. Intracortical inhibition (ICI) and Intracortical facilitation (ICF) were studied using the conditioning-test paradigm applying two magnetic stimuli in rapid succession [22], and the effect of the first (conditioning) stimulus on the second (test) stimulus was investigated. The procedure was performed over the primary motor cortex through the same stimulating coil deriving pulses from two Magstim 200 Stimulators, connected via a Bistim module (The Magstim Company, Whitland, Dyfed, UK) connected to a CED micro 1401 interface (Cambridge Electronic Design, Cambridge, UK). The conditioning stimulus was set at 80% of the subjects rMT whereas the test stimulus at 130% of the rMT. The interstimulus intervals (ISIs) tested were 2, 3, 10 and 15 ms. Ten trials for each ISI were recorded in a random way. The responses were expressed as the ratio between the MEP amplitude produced by paired stimulation and that produced by test stimulus alone.

The subjects were seated in a comfortable chair with continuous EMG monitoring to ensure either a constant level of EMG activity during tonic contraction or complete relaxation at rest. Data were collected on a computer and stored with an ad hoc software for off-line analysis [23]. All TMS procedures were performed in the same laboratory and experimental conditions, at the same time of the day and by the same operators, thus minimizing the inter-subject variability.

### Statistical analysis

The comparison of the frequency of observation of some clinical features was carried out by means of the Chi-square test. Because of the relatively low number of subjects and the non-normal distribution of data (as determined by the Shapiro-Wilk’s test of normality), all the other comparisons were performed by means of the non-parametric Wilcoxon test for paired datasets. Differences were considered significant a p<0.05 level. However, because of the relatively limited number of subjects available and to rule out possible type II errors, we also calculated effect sizes using the Cohen’s d value [24]. Cohen’s d is defined as the difference between two means divided by their pooled standard deviation. According to Cohen, 0.2 is indicative of a small effect, 0.5 of a medium and 0.8 of a large effect size.

## Results

As shown in Table 2, no difference was found for clinical features between the initial and the follow-up evaluation. Global cognitive functioning as assessed by MMSE did not change significantly over time. The HDRS score showed a trend towards an improvement at follow-up (with a moderate effect size); a dysthymic disorder, evaluated by the SCID-I, was found in only one patient at follow-up, vs. 5 at baseline.

**Table 2:** Clinical features of the CD subjects at follow-up.

	Baseline (n = 13)		Follow-up (n = 13)		Wilcoxon	Effect Size
	*median*	*25* ^*th*^ *-75* ^*th*^ *quartile*	*median*	*25* ^*th*^ *-75* ^*th*^ *quartile*	*p*	*Cohen’s d*
MMSE	30.0	29.0–30.0	30.0	30.0–30.0	NS	-0.495
HDRS	8.0	3.0–10.0	3.0	0.0–5.0	*0*.*083*	0.687
					**Chi-square**	
	Yes/No		Yes/No		*p*<*	
Neurologic signs	2/11		2/11		NS	
Comorbidity	10/3		7/6		NS	
Dysthymia (SCID-I)	5/8		1/12		*0*.*063*	

CD = celiac disease; MMSE = Mini Mental State Examination; HDRS = 17 item-Hamilton Depression Rating Scale; SCID-I = Structured Clinical Interview for DSM-IV Axis I.

**Fig 1:**
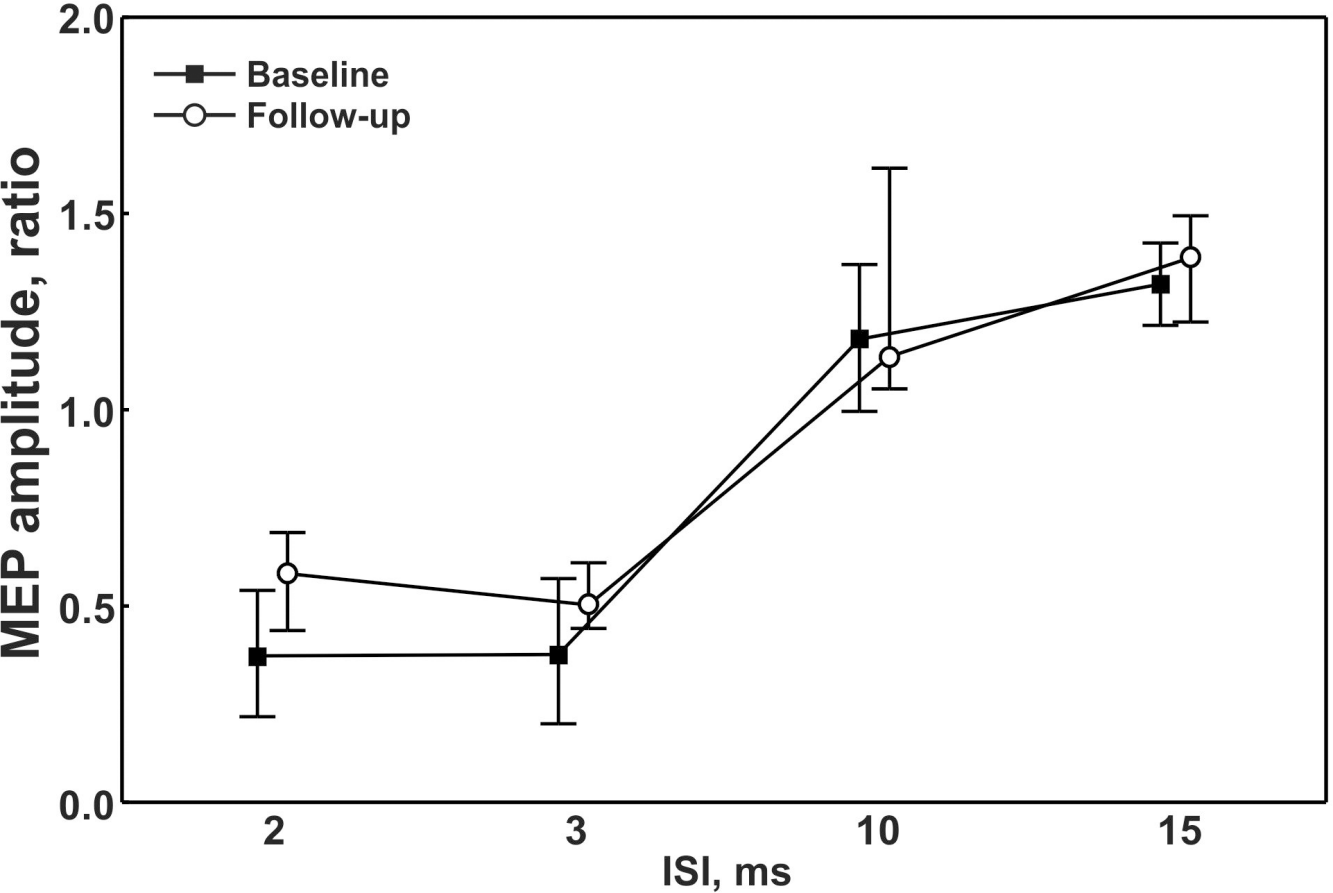
Intracortical excitability at different interstimuls intervals at baseline and follow-up. ISI = interstimuls interval; MEP = Motor Evoked Potential.

Compared to baseline, treated patients showed a statistically significant decrease of the median rMT values (from 35% down to 33%, p = 0.0042). The other single-pulse TMS measures (Table 3), as well as the paired-pulse TMS parameters (ICI and ICF), did not change significantly after the follow-up period (Fig 1), thus showing persistent disinhibition and hyperfacilitation. Maximal M-waves were comparable between sessions. Examination of the individual data taking into account the differences of the rMT values from baseline (ΔrMT) and the length of the follow-up showed additional information. Specifically, after a longer follow-up period, 4 out of 6 patients with serological conversion had the largest ΔrMT whereas, at shorter time of follow-up, 5 out of 7 patients with antibodies persistence exhibited the smallest ΔrMT. The small number of patients did not allow a more detailed statistical analysis of this aspect.

**Table 3:** Comparison of electrophysiological data.

	Baseline (n = 13)		Follow-up (n = 13)		Wilcoxon	Effect Size
	*Median*	*25* ^*th*^ *-75* ^*th*^ *quartile*	*Median*	*25* ^*th*^ *-75* ^*th*^ *quartile*	*p*	*Cohen’s d*
**rMT, %**	35.0	34.0–41.0	33.0	29.0–37.0	0.0042	0.472
**CSP, ms**	100.0	76.0–112.0	83.0	57.0–128.0	NS	-0.090
**MEP latency, ms**	18.4	18.3–20.2	18.9	17.9–20.6	NS	-0.118
**CMCT, ms**	5.6	4.3–6.3	5.2	5.0–5.7	NS	0.199
**CMCTF, ms**	4.8	4.5–5.5	5.2	4.7–5.6	NS	-0.161
**A ratio**	0.3	0.2–0.6	0.3	0.2–0.4	NS	-0.234
**F amplitude, μV**	0.1	0.1–0.2	0.2	0.1–0.2	NS	-0.192

rMT: resting motor threshold; CPS: cortical silent period; MEP: motor evoked potentials; CMCT: central motor conduction time; CMCTF: central motor conduction time estimated by F wave; A ratio: amplitude ratio.

## Discussion

To our knowledge, this is the first prospective study exploring the impact of the gluten-free diet on cortical excitability to TMS in CD patients. The main result is that, after the gluten-free diet, a global increase of cortical excitability, as indexed by a reduction of the median rMT, was observed, without any significant changes of the other TMS measures. As known, the rMT is a global measure of motor system excitability and is thought to reflect mainly the membrane excitability of the cortical-spinal neurons and the glutamatergic excitatory interneurons that project into these neurons [25,26].

Although the reason underlying this change is rather complex, it is known that the net effect of increased excitation could be the result of prevailing activation and/or weaker inhibition, phenomena involving different neurochemical pathways. In this context, it is worth to note that both the CSP shortening and the reduced ICI did not change over time, suggesting the persistence of a GABA-mediated intracortical dysfunction despite the dietary restriction [5]. Accordingly, the hyperexcitability might be the result of an immune-mediated dysregulation of inhibitory GABAergic interneurons or a parallel glutamate-mediated cortical functional reorganization compensating for disease progression despite the adoption of the gluten free-diet. In particular, the AMPA type ionotropic glutamate receptor is known to be crucial for plasticity and excitatory synaptic transmission at the level of many post-synaptic membranes [27], including the motor cortex [28]. Thus, it cannot be excluded that receptor activation, probably triggered by CD-related immune system dysregulation, might result in a long-lasting increase of excitatory post-synaptic potentials underlying the observed motor cortex hyperexcitability and probably related to phenomena of long-term plasticity. Although obtained from a small number of patients, the analysis of individual data seems to reveal a correlation between the ΔrMT, the serological status and the length of the gluten-free diet. In particular, even if a median decrease of the rMT was globally observed in all participants, a relatively greater hyperexcitability occurred in those with seroconversion and a longer gluten restriction. This might suggest a more powerful involvement of hyperexcitability-induced cortical plasticity in these patients, as demonstrated in other neurological disorders by specific TMS mapping techniques [29,30].

The lack of studies assessing TMS parameters in newly diagnosed CD does not allow to compare our data with other cohorts and very few studies have explored subclinical neurophysiology of CD after alimentary therapy. In 27 asymptomatic children on gluten-free diet, Cakir et al. found 2 of them with EMG-documented peripheral polyneuropathy, one with prolonged central conduction time at somatosensory evoked potentials and 3 with a single seizure but normal EEG, indicating that subclinical neurological abnormalities may persist despite the diet [31]. More recently, Parisi et al. revealed subclinical EEG abnormalities in 48% of children, which disappeared in 78% of them after 6 months of dietary restriction, suggesting that cortical excitability in asymptomatic children with newly diagnosed CD is modified by the introduction of the diet [32].

Notably, the role of the gluten-free diet in subclinical neurological abnormalities has not been assessed and the impact on the histologic course needs to be further investigated. It is well established that the gluten-free diet represents the most important aspect of the management of CD patients and it is the only treatment that allows the prevention of several associated malignant and non-malignant complications, including neurological diseases. Histologic recovery of the small intestinal mucosa is assumed to occur within 6–12 months after starting a gluten restriction, simultaneously with clinical remission. However, follow-up data on intestinal recovery in CD patients are scarce and contradictory, even in patients with a good diet compliance [33–36]. In a long-term follow-up study of 158 patients, the histologic recovery in gluten-sensitive sprue after starting a gluten-free diet took more than 2 years in 35.4% of patients and was incomplete or absent in a substantial subgroup of patients [37].Other studies reported that neurological damage or antibodies persisted despite the strict adherence to the diet, showing the ineffectiveness of a gluten-free diet in obtaining neurological remission in CD [38–41]. It is noteworthy that 7 of our patients did not achieve serological conversion. Furthermore, neurological deficits may even develop despite strict adherence to a gluten-free diet, as shown in different studies [38–42].

Based on these considerations, it can be argued that the length of the follow-up period may be insufficient for gluten-associated conditions to recover and for CD antibodies to disappear. Indeed, current knowledge on the efficacy of the gluten-free diet in the natural course of related neurological complications seems to depict a first step when they are still gluten-sensitive and a following step which is considered to be gluten insensitive [38]. The high mean age at diagnosis of our patients or, in other words, the long-standing gluten exposure may explain why subclinical neurological involvement persisted throughout the follow-up. This hypothesis is supported by previous CD-associated epilepsy studies suggesting that the efficacy of the diet is inversely related to the duration of neurological disorders as well as to the age of the patient at the start of the gluten-free diet [32,43].

Finally, in this study, a certain degree of improvement of depressive symptoms was also observed, supporting the role of the gluten-free diet in the amelioration of psychiatric CD-related disorders. Recognizing depression as a risk factor for a poor adherence to the gluten-free diet is of great importance. Consequently, it would be advisable to consider, both at the diagnosis and at follow-up, not only clinical and serological features, but also the presence of depression, so that psychological support or pharmacological therapy might be started [4,44].

There are some limitations to take into account when interpreting the findings of this study. First, as usual in TMS research, the relatively small number of patients requires further independent investigations with larger series of participants. In addition, future studies on CD patients with a long-term dietary restriction will further clarify the meaning of the detected changes and their temporal relation with the gluten-free diet duration and compliance. Second, the reliability of the rMT measurements over time is of crucial importance given that there was no untreated control group. For instance, the hypothesis that the observed increase of motor cortex excitability could be related to aging should be considered. However, most TMS studies have not found differences in the primary motor cortex excitability threshold between young and elderly [45–48], whereas others have shown an increase in motor threshold in healthy subjects with aging [49,50]. Conversely, our patients showed a significant decrease of the median rMT value at follow-up, indicating a higher excitability. Regarding the test-retest reliability of rMT measured at baseline and repeated at follow-up, most TMS studies converge on the evidence that rMT is reliable when measured over time, in both healthy subjects [51,52] and patients with neurological disorders such as stroke [53–55]. However, as the effect of the gluten-free diet on the rMT was relatively small (35% *vs* 33%), the observed change needs to be considered in view of a number of issues pertaining to reliability of repeated cortical-motor threshold measurements (such as the TMS coil placement, the arm and electrode positioning). Therefore, this finding might not be large enough to uphold the conclusion of increased cortical excitability, although multiple comparisons have been made increasing the likelihood of a significant result. Finally, the timing of testing after starting the gluten-free diet was not uniform. Nevertheless, given that, as known, complete healing of the small intestine after the adoption of the gluten-free diet may take longer than 12 months (even more than 24 months in some cases), we established to re-evaluate these patients after at least one year of adequate gluten restriction.

In conclusion, in this study we showed that the gluten-free diet modulated the TMS indexes of cortical function in adult CD patients. TMS, integrated with clinical, immunological and advanced neuroimaging data, might be considered as an additional tool for evaluating the progression of neurological involvement in CD and the response to the dietary regimen.
